# Genetic Evidence That Captured Retroviral Envelope *syncytins* Contribute to Myoblast Fusion and Muscle Sexual Dimorphism in Mice

**DOI:** 10.1371/journal.pgen.1006289

**Published:** 2016-09-02

**Authors:** François Redelsperger, Najat Raddi, Agathe Bacquin, Cécile Vernochet, Virginie Mariot, Vincent Gache, Nicolas Blanchard-Gutton, Stéphanie Charrin, Laurent Tiret, Julie Dumonceaux, Anne Dupressoir, Thierry Heidmann

**Affiliations:** 1 Unité Physiologie et Pathologie Moléculaires des Rétrovirus Endogènes et Infectieux, CNRS UMR 9196, Institut Gustave Roussy, Villejuif, France; 2 Université Paris-Sud, Orsay, France; 3 UPMC Université-Paris 6, UM 76, Paris, France; 4 INSERM U974, Paris, France; 5 CNRS UMR 7215, Paris, France; 6 Institut de Myologie, Paris, France; 7 INSERM IMRB U955-E10, Créteil, France; 8 Université Paris-Est, Ecole nationale vétérinaire d'Alfort, Maisons-Alfort, France; 9 Université Paris-Est Créteil, Faculté de médecine, Créteil, France; 10 INSERM U935, Villejuif, France; 11 Université Paris-Sud, Institut André Lwoff, Villejuif, France; The Jackson Laboratory, UNITED STATES

## Abstract

*Syncytins* are envelope genes from endogenous retroviruses, “captured” for a role in placentation. They mediate cell-cell fusion, resulting in the formation of a syncytium (the syncytiotrophoblast) at the fetomaternal interface. These genes have been found in all placental mammals in which they have been searched for. Cell-cell fusion is also pivotal for muscle fiber formation and repair, where the myotubes are formed from the fusion of mononucleated myoblasts into large multinucleated structures. Here we show, taking advantage of mice knocked out for *syncytins*, that these captured genes contribute to myoblast fusion, with a >20% reduction in muscle mass, mean muscle fiber area and number of nuclei per fiber in knocked out mice for one of the two murine *syncytin* genes. Remarkably, this reduction is only observed in males, which subsequently show muscle quantitative traits more similar to those of females. In addition, we show that *syncytins* also contribute to muscle repair after cardiotoxin-induced injury, with again a male-specific effect on the rate and extent of regeneration. Finally, *ex vivo* experiments carried out on murine myoblasts demonstrate the direct involvement of *syncytins* in fusion, with a >40% reduction in fusion index upon addition of siRNA against both *syncytins*. Importantly, similar effects are observed with primary myoblasts from sheep, dog and human, with a 20–40% reduction upon addition of siRNA against the corresponding *syncytins*. Altogether, these results show a direct contribution of the fusogenic *syncytins* to myogenesis, with a demonstrated male-dependence of the effect in mice, suggesting that these captured genes could be responsible for the muscle sexual dimorphism observed in placental mammals.

## Author Summary

Syncytins are “captured” genes of retroviral origin, corresponding to the fusogenic envelope gene of endogenized retroviruses. They are present in all placental mammals in which they have been searched for, where they play an essential role in placentation via their cell-cell fusion activity. Here we show that they also contribute to myoblast fusion and muscle formation in development and repair after injury, using both *in vivo* knock-out mouse models and *ex vivo* primary myoblast cell cultures from several mammals, including humans, carnivores and ruminants. Interestingly, the effects observed in mice are sex-dependent, thus suggesting that the added “collateral” effect of syncytins on myogenesis could be responsible for the muscle sexual dimorphism observed in placental mammals.

## Introduction

*Syncytins* are “captured” genes of retroviral origin that correspond to the envelope gene of ancestrally endogenized retroviruses (reviewed in [1]). These genes encode fusogenic proteins that are involved in the formation by cell-cell fusion of the placental syncytiotrophoblast in eutherian mammals and marsupials [2–5]. Furthermore, genetically modified mice, knocked out for their two *syncytin* genes–i.e. *syncytin-A* and *syncytin-B* [6]—proved to be deficient in placenta development, with altered structures of the materno-fetal interface resulting, in the case of Syncytin-A, in the death of the embryo at mid-gestation [7, 8]. It thereafter turned out that *syncytins* can be found in all placental mammals in which they had been searched for, with independently captured *syncytins* found in all major clades of placental mammals, including the Euarchontoglires (primates, rodents, lagomorpha), Laurasiatherians (ruminants, carnivores), Afrotherians (tenrec), and even the marsupials (opossum). This has led to the hypothesis that these genes, which are absolutely required for placentation, as shown by the knock-out mice experiments, are most likely responsible for the emergence of placental mammals from egg-laying animals. Analysis of the conservation of these “new” genes clearly indicated that they have been subjected to purifying selection in the course of evolution, as expected for any bona fide cellular gene. Of note, cell-cell fusion is a basic phenomenon for all species, from drosophila to humans, where such events are involved in numerous processes including, in addition to placentation in mammals, macrophage fusion for osteoclast formation, and most importantly myoblast fusion for myogenesis during development and for muscle regeneration after injury (reviewed in ref. [9–13]). Although *syncytins* are essentially expressed in the placenta trophoblast cells, low level expression can be detected in other cells such as fusing macrophages [14] and myoblasts [15], and we therefore tested, using genetically modified mice, whether *syncytins* could participate in muscle formation, thus revealing a “collateral” effect of the “primary” capture of these genes for placentation.

Myoblast fusion is a complex and tightly controlled process required for the formation of the skeletal muscle fibers (reviewed in refs. [9, 10, 16]). The fusion process is highly cell-type specific to ensure that fusogenic myoblasts do not form syncytia with non-muscle cells. The molecular mechanisms that coordinate myoblast fusion remain incompletely understood, although several partners of myoblast fusion have already been identified. These include numerous proteins involved in cell-cell adhesion, as well as proteins involved in actin and lipid dynamics that allow cytoskeletal and membrane remodeling for cell fusion. Furthermore, a muscle-specific membrane protein called myomaker that is transiently expressed during myogenesis and is both necessary and sufficient to promote myoblast fusion *in vivo* and *in vitro* has recently been identified [17, 18]. Although this protein alone is not sufficient to induce the fusion of non-myoblast cells such as fibroblasts, myomaker is likely to be a major player of the membrane fusion process; yet its partner (either a receptor or a bona fide fusogenic ligand) remains to be identified. Altogether, the numerous studies carried out on myoblast fusion have led to the conclusion that these processes involve numerous partners and pathways. As syncytins have been shown to be bona fide fusogens, we investigated their involvement in the overall myogenesis process.

## Results

### *Syncytin-B* knockout male mice present a delay in muscle development

**Fig 1:**
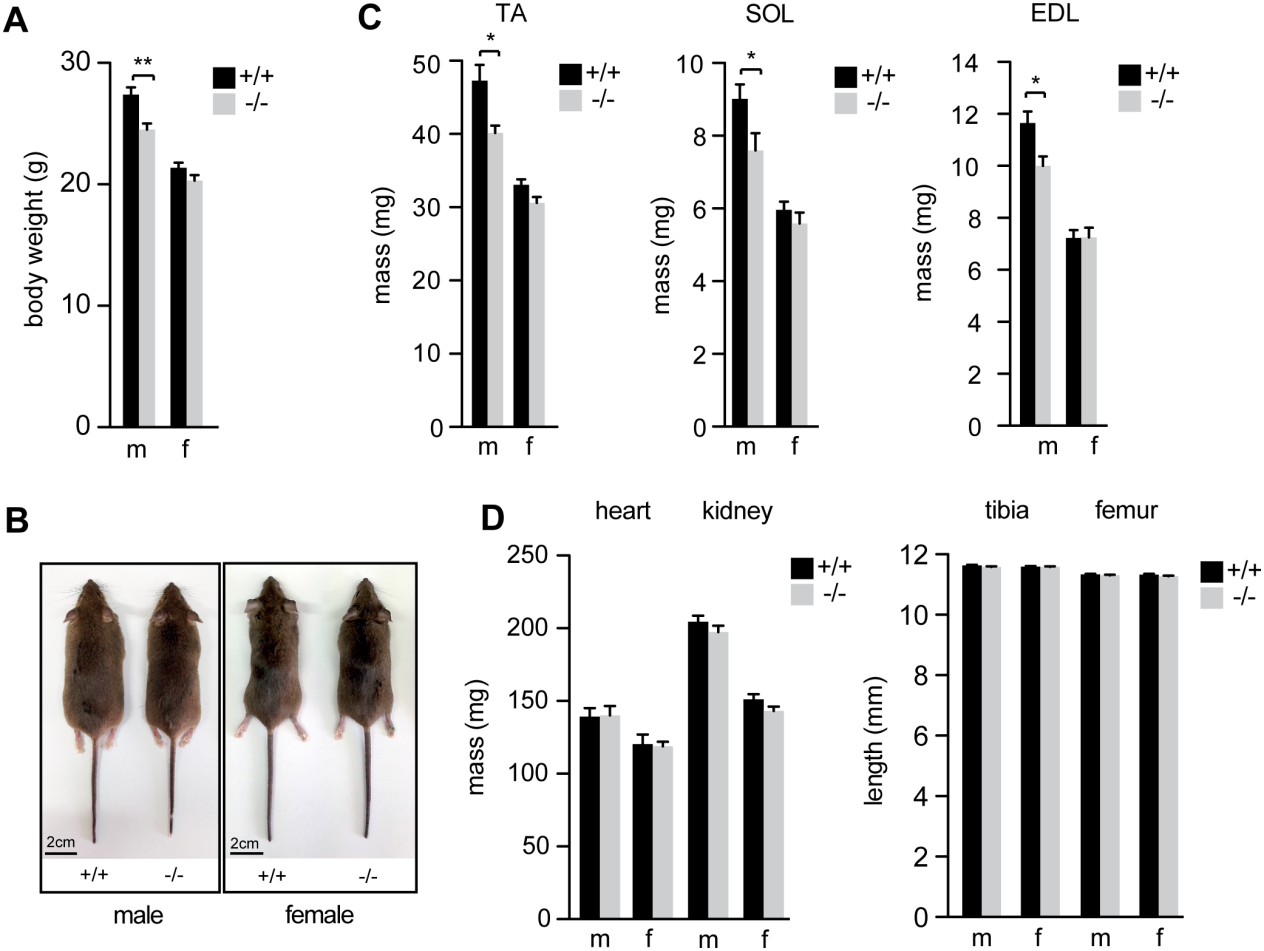
Phenotype of 12 week old SynB^-/-^ and WT mice. (*A*) Body weight. (*B*) Representative WT and SynB^-/-^ male (m) and female (f) mice. (*C*) Muscle mass of three skeletal muscles located in the hindlimb, TA (*Tibialis Anterior*), SOL (*Soleus*), EDL (*Extensor Digitorum Longus*). (*D*) Mass of heart and kidney, length of tibia and femur. Data are the mean ± SEM (number of mice per sex and per genotype: 13–17 in *A*, 5–11 in *C*, 4–7 in *D*; * p<0.05, ** p<0.01, Mann and Whitney test).

As previously reported, whereas deletion of both *syncytin* genes, i.e. *syncytin-A* and *syncytin-B* leads to embryonic lethality, knockout of *syncytin-B* alone allows for surviving progeny to be obtained. Yet, *syncytin-B* null (SynB^-/-^) neonates display a sex-independent growth retardation characterized by a reduction of body weight of 18% when compared to wild-type neonates from the same litter [8]. This difference was attributed to associated placental malformations and was still apparent in 6–8 week old male mice but absent in female mice [8]. In order to clarify whether the observed decrease in body weight of male mice could be attributed to a reduction in muscle mass, we measured, in adult 12 week old mice, both the body weight (Fig 1A and 1B) and the mass of several skeletal muscles, located in the hind limbs of the mouse: two fast-twitch muscles, i.e. *Tibialis Anterior* (TA) *and Extensor Digitorum Longus* (EDL), and one slow-twitch muscle the *Soleus* (SOL) (Fig 1C). Measurements were performed on F1 mice, either wild-type or SynB^-/-^, from at least four different crosses between heterozygous SynB^+/-^ mice, in order to minimize possible lineage effects and compare genetically related individuals. As shown in Fig 1A and 1C, adult SynB^-/-^ and WT female mice show no differences in body weight and muscle mass (17 wt and 13 SynB^-/-^ females). However, adult SynB^-/-^ male mice show a significant reduction in body mass (12%; p<0.01, Mann and Whitney test; 16 wt and 16 SynB^-/-^ males). This decrease is associated with a reduction in muscle mass as compared to wild type male mice (15%; p<0.05, Mann and Whitney test), which is observed independently of the crossing (S1A Fig). We also quantified, in the corresponding animals, as an internal control, the mass of two organs (heart and kidney), and the length of two bones (tibia and femur), with no significant differences seen between SynB^-/-^ and WT mice (Fig 1D). These last results suggest that the reduction in body weight in adult SynB^-/-^ male mice is mainly due to a decrease in muscle mass (which accounts for 35–40% of the body weight). In addition, these decreases appear to affect only skeletal muscles, where fusion processes take place. Altogether these results suggest that Syncytin-B takes part into muscle development in male mice.

### *Syncytin-B* knockout male mice exhibit a reduction in myofiber size and myonuclei number per fiber

**Fig 2:**
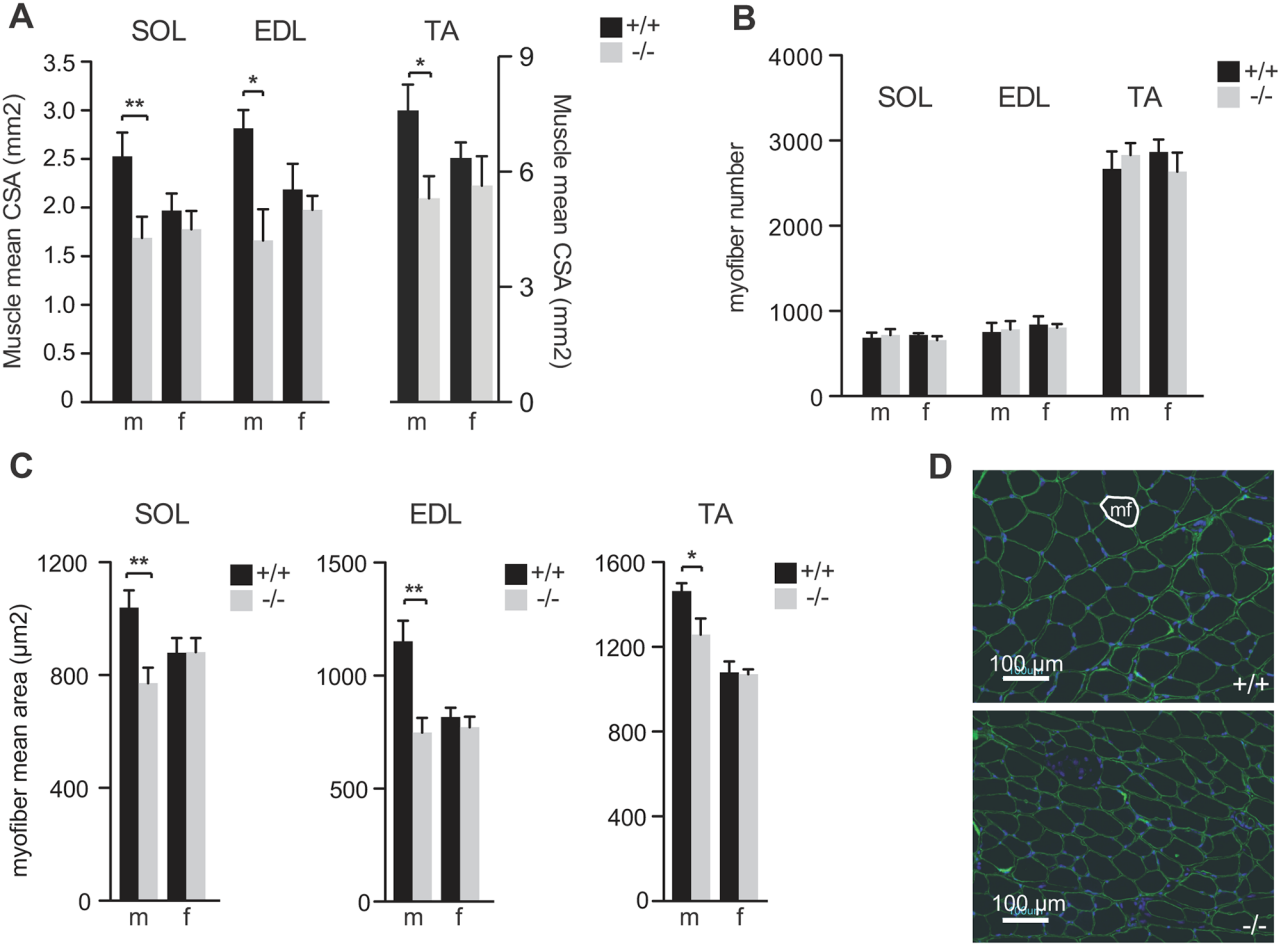
Muscle phenotype of 12 week old SynB^-/-^ mice. (*A*) Cross-sectional area (CSA) of SOL, EDL and TA muscles (mm^2^) of WT and SynB^-/-^ male and female mice. (*B*) Number of myofibers per muscle section. (*C*) Myofiber CSA (μm^2^) in SOL, EDL and TA muscles of WT and SynB^-/-^ male and female mice. Histological analysis was performed on at least 150 myofibers randomly chosen from three different tissue sections for each condition. (*D*) Anti-dystrophin and DAPI labelling of 10 μm sections of optimum cutting temperature (OCT)-frozen EDL muscles from 12 week old male mice (scale bar: 100 μm). A myofiber (mf) is delineated in white. Data are the mean ± SEM (5–8 mice analyzed per sex and per genotype; * p<0.05, ** p<0.01, Mann and Whitney test).

**Fig 3:**
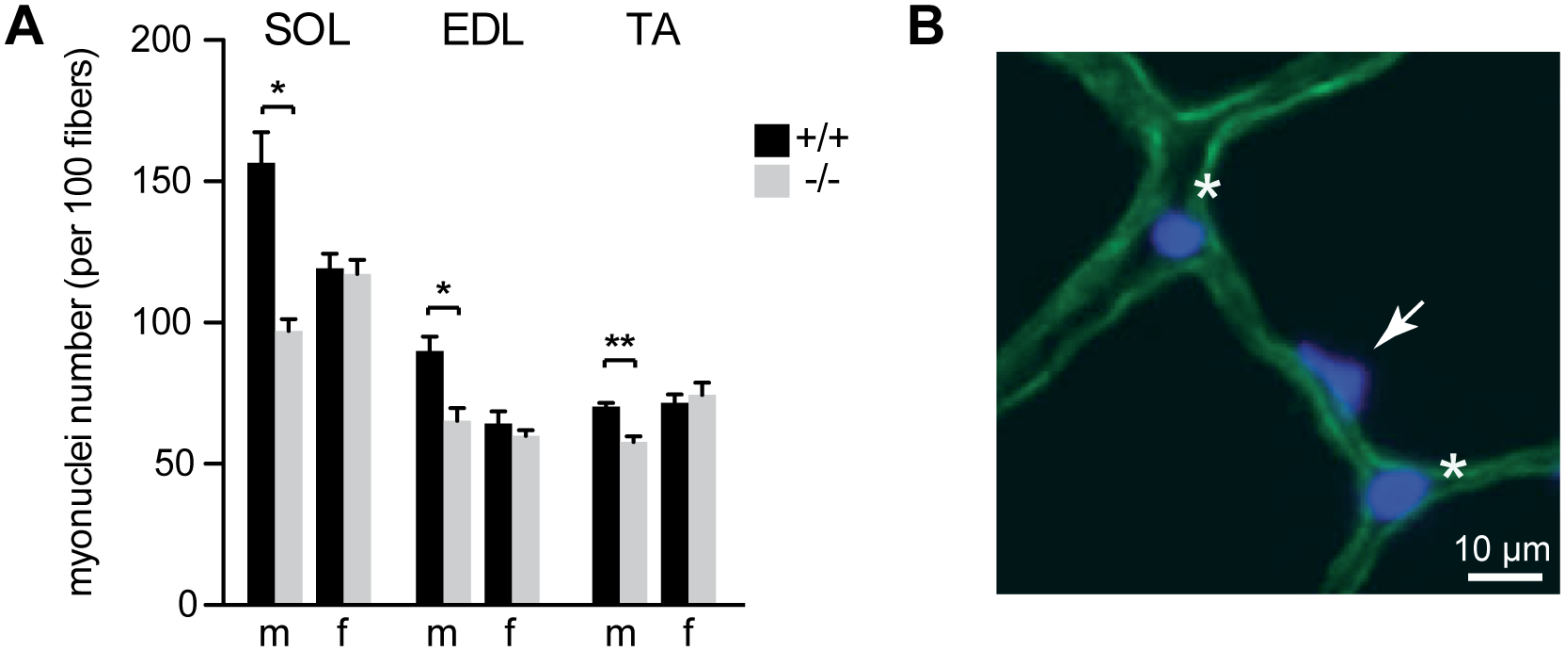
Myonuclei number in 12 week old SynB^-/-^ mice. (*A*) Quantification of the myonuclei number per 100 fibers in SOL, EDL and TA muscles of WT and SynB^-/-^ male and female mice. The number of myonuclei was estimated by analyzing at least 300 myofibers for each condition. Data are the mean ± SEM (4–6 mice analyzed per sex and per genotype; * p<0.05, ** p<0.01, Mann and Whitney test). (*B*) Nuclei corresponding to myonuclei (white arrow) are located within the myofibers. Nuclei outside myofibers (white asterisk), including those from satellite cells, were not counted.

To better characterize the role of *syncytin-B* in muscle development, morphological analyses of SynB^-/-^ and WT muscles (see scheme in S2 Fig) were performed on cryosections of TA, EDL and SOL muscles, respectively. We first quantified the cross-sectional area (CSA) of the entire muscle, which was found to be reduced for the three muscles in SynB^-/-^ males as compared to WT males (Fig 2A; * p<0.05, ** p<0.01, Mann and Whitney test). The alteration in muscle CSA was observed independently of the crossing (four crosses at least) (S1B Fig). As expected, in females, which did not show a reduction in muscle mass, no differences in CSA were observed between SynB^-/-^ and wild-type mice. The number of fibers per muscle and the individual myofiber CSA, both parameters that contribute to muscle CSA, were also measured, as illustrated in Fig 2. Equivalent fiber numbers were observed between SynB^-/-^ and wild-type male mice (Fig 2B), but a significant reduction in myofiber CSA was observed in SynB^-/-^ male mice (Fig 2C and 2D; * p<0.05, ** p<0.01, Mann and Whitney test). The alteration in myofiber mean area was observed independently of the crossing (three to four crosses) (S1C Fig). Finally, we quantified the number of myonuclei per fiber, in knockout and wild-type mice (Fig 3). Transversal sections of the three muscles TA, EDL and SOL were immunolabeled with a dystrophin antibody to outline the sarcolemma, and were stained with 4',6'-diamidino-2-phenylindole (DAPI) to visualize the nuclei. Nuclei located inside the dystrophin-labeled sarcolemna were identified as myonuclei and counted (Fig 3B). Accordingly, a decrease of approximately 30% in the myonuclei number was observed in muscles from SynB^-/-^ male mice as compared to control males (Fig 3A; * p<0.05, ** p<0.01, Mann and Whitney test), strongly suggestive of a defect in myoblast fusion. This difference was not observed in SynB^-/-^ females in comparison to wild-type. As variations in nuclear size and shape may affect myonuclear count [19], we measured the nuclear length of myonuclei on longitudinal sections. No differences were observed between either WT or SynB^-/-^, male or female mice (S3A and S3B Fig). As expected, the resulting number of myonuclei per mm of fiber (S3C–S3E Fig) showed a decrease in muscles from SynB^-/-^ males as compared to control males, whereas no significant differences were observed in females. Since myoblast fusion and accumulation of myonuclei contribute to mammalian myofiber development and growth [20–22] the data also strongly suggest that the reduced myofiber CSA observed in SynB^-/-^ male mice, which have an unaltered myofiber number, mainly results from a defect in myoblast fusion to myofibers.

### *Syncytin-B* plays a role during muscle regeneration in male mice

**Fig 4:**
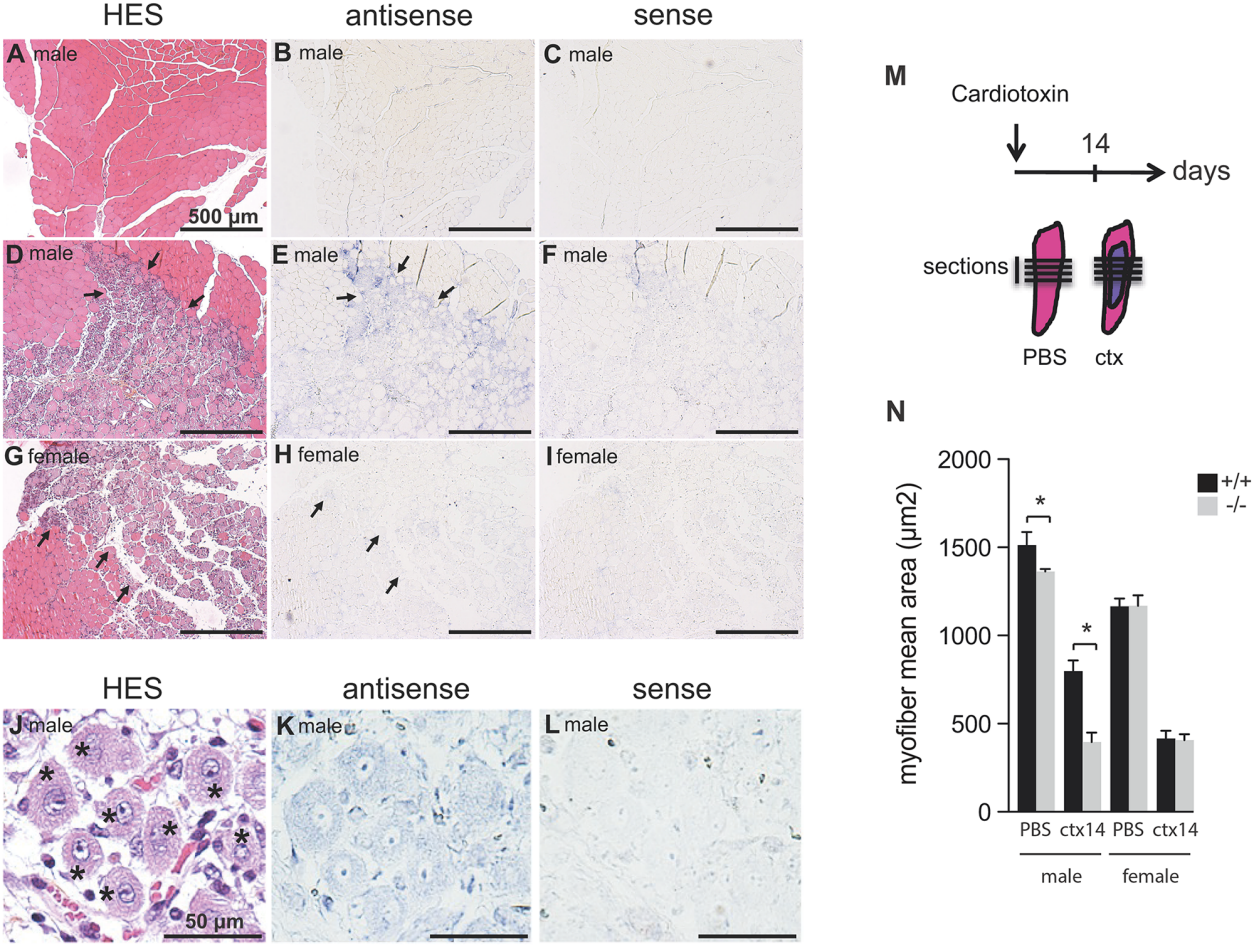
Impact of syncytin-B during muscle regeneration. (*A-L*) *In situ* hybridization of *syncytin-B* during muscle regeneration following cardiotoxin injury. Mice were injected with PBS or with cardiotoxin (Ctx) 4 days before muscle examination. Sections were stained with HES (*A*, *D*, *G* and *J*) and were hybridized with a sense probe (*C*, *F*, *I* and *L*) or with an antisense probe specific of *syncytin-B* (*B*, *E*, *H* and *K*). The regenerative zone of the muscle section is indicated by black arrows (*D*, *E*, *G* and *H*). The regenerative zone is composed of small centro-nucleated fibers, indicated by asterisks (*J*). (*A*-*I*) scale bar 500 μm; (*J*-*L*) scale bar 50 μm. *(M*, *N)* 8 week old WT and SynB^-/-^ male and female mice were injected with PBS (control) or cardiotoxin (Ctx) and muscle regeneration was characterized at day 14. Histological analysis was performed on at least 150 myofibers randomly chosen from three different tissue sections per condition. Data are the mean ± SEM (4–5 mice analyzed per sex and per genotype; * p<0.05, Mann and Whitney test). When measuring myofiber mean area of SynB^-/-^ in comparison to wild-type male mice after Ctx treatment, a delay is clearly visible at day 14 of regeneration. Of note, the difference observed after control PBS injection is consistent with that observed in Fig 2C for uninjured animals.

Muscle regeneration is a physiological process that recapitulates, through the differentiation and fusion of satellite cell descendants, all the phases of myogenesis [16]. We therefore investigated the requirement for *syncytin-B* during muscle regeneration. We first analyzed, by *in situ* hybridization, the expression of *syncytin-B* upon muscle injury. Muscle injury was induced by a single injection of cardiotoxin into the TA muscle of 8 week old female and male mice. Injection of PBS in the contralateral muscle was used as a control. Muscles were collected after 4 days of regeneration (see scheme in Fig 4M). Hematoxylin, eosin and safran staining (HES) of the muscle sections (Fig 4A), shows normal myofibers after PBS injection, with myonuclei located at the myofiber periphery. In contrast, following cardiotoxin injection, similar damage of the muscle section could be observed (delineated by black arrows, Fig 4D and 4G) in male and female mice. During muscle regeneration, the damaged myofibers are destroyed and replaced by small newly centro-nucleated fibers, typically representative of muscle regeneration. These new fibers observed in male as in female mice, are illustrated in the enlarged Fig 4J for a male mouse. Specific digoxigenin-labeled antisense probes were then synthetized for the detection of *syncytin-B*, and the corresponding sense probes were used as negative controls. As further shown in Fig 4E and 4K, labeling was only observed with the antisense probe for *syncytin-B*, and only in males. Closer examination of the labeling further shows that it is restricted to regions of muscle regeneration (Fig 4D and 4E) where myofibers are centro-nucleated (Fig 4J and 4K). *In situ* hybridization of *syncytin-B* shows conclusively that this gene is expressed in the regenerative muscle in males, strongly suggesting that the encoded protein could play a role in this process specifically in males.

To characterize further the role of the Syncytin-B protein in this process, SynB^-/-^ male and female mice (as well as control wild-type mice) were analyzed after cardiotoxin injection as above, and muscle regeneration was quantified at day 14. Histological sections of the muscle fibers were performed as above, and the mean myofiber CSA measured. As illustrated in Fig 4N, quantification of the myofiber CSA in SynB^-/-^ male mice in comparison to wild-type shows a delay at day 14, suggesting that Syncytin-B plays a role during muscle regeneration (Fig 4N; p<0.05, Mann and Whitney test) after cardiotoxin treatment. In the case of female mice, no differences could be detected in the myofiber CSA between SynB^-/-^ and wild-type, suggesting that Syncytin-B plays a role during muscle regeneration also in a sex-dependent manner (Fig 4N).

### *Syncytin-A* and *-B* are involved in myoblast fusion

**Fig 5:**
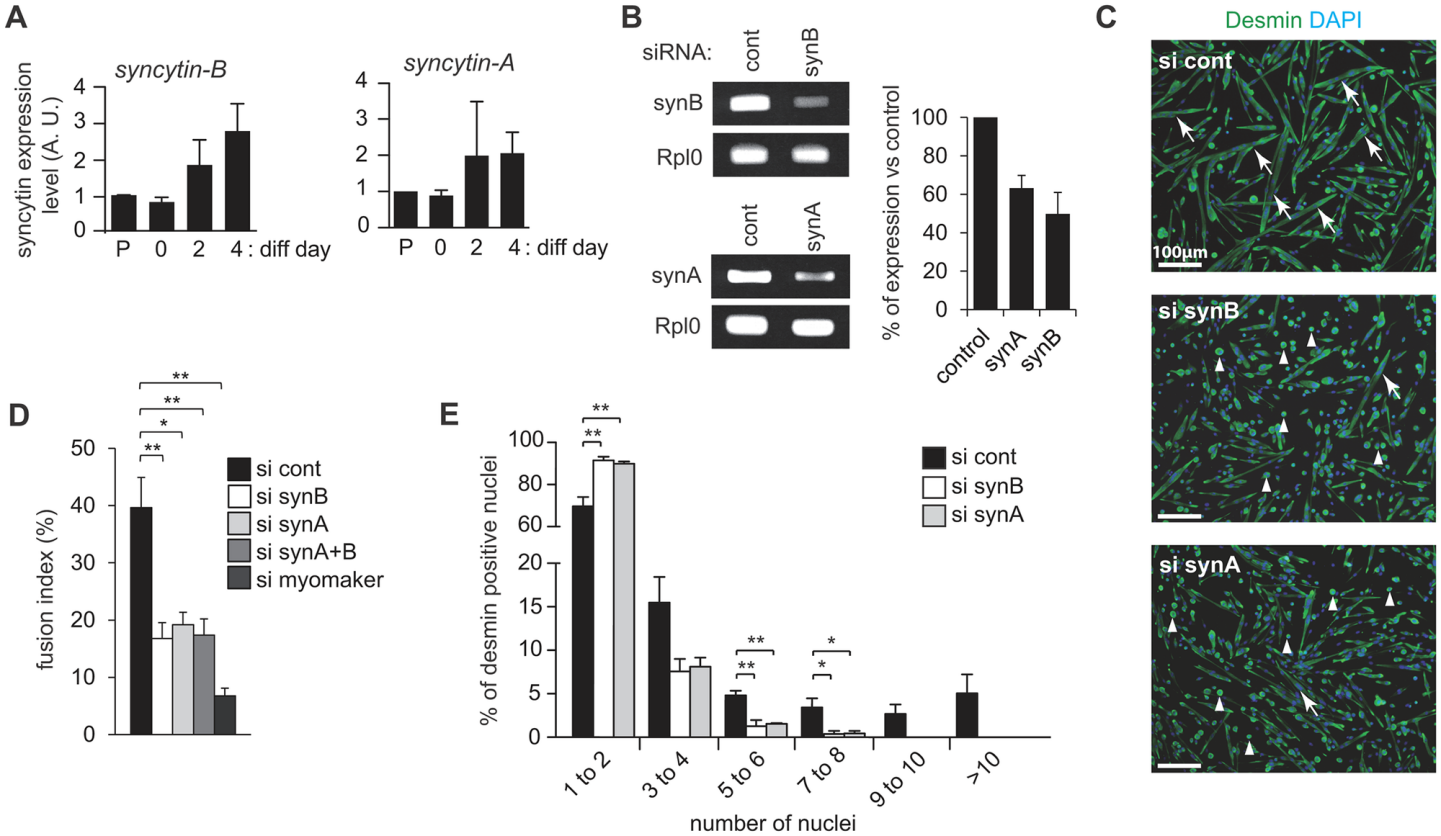
Murine syncytin expression and impact of their knockdown on myoblast cell-cell fusion *ex vivo*. Primary myoblast cells were transfected with control siRNA (si cont) or siRNAs specifically designed against *syncytin-B* (si synB),–*A* (si synA) or myomaker (si myomaker), and differentiation was induced 2 days later. Following two days of differentiation, cells were fixed in 4% PFA, stained with an anti-desmin antibody (muscle cell marker), and nuclei were counterstained with DAPI. (*A*) Level of expression of *syncytin-B* and *-A* (arbitrary units, A. U.) during myoblast fusion in murine primary myoblasts, either actively proliferating (P: proliferating non confluent cells, D0: proliferative confluent cells), or in differentiation and fusion (D2 to D4), as analyzed by quantitative RT-PCR. All quantifications were normalized by RPL0, and the fold-change in gene expression expressed relative to the values of proliferating myoblasts, arbitrarily settled to one. (*B*) Efficiency of siRNA knockdown measured by quantitative RT-PCR. (*C*) Representative images of desmin- and DAPI-labelled myoblast cells transfected with control, *syncytin-B* or *syncytin-A* siRNAs. Representative myotubes are indicated by white arrows whereas white triangles correspond to mononucleated myoblast cells. (*D*) Fusion index (number of nuclei in cells with ≥2 nuclei per total number of nuclei). (*E*) Nuclei distribution per myotube. Data are the mean ± SEM (four independent experiments; * p<0.05, ** p<0.01, Mann and Whitney test).

**Fig 6:**
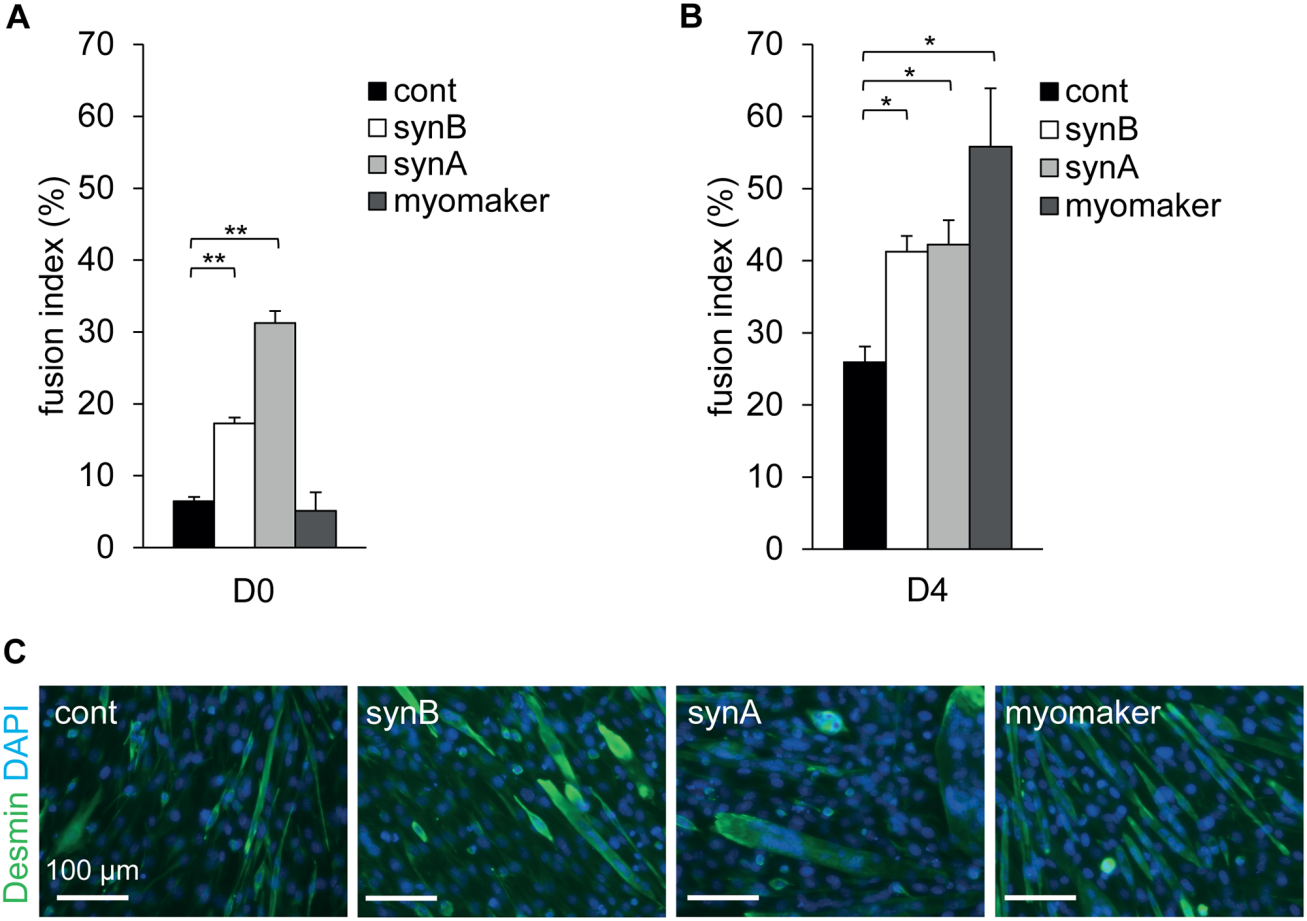
Impact of murine syncytins and myomaker overexpression on myoblast cell-cell fusion *ex vivo*. C2C12 myoblasts were co-transfected with an empty (cont), syncytin-A (synA),–B (synB) or myomaker expression vector, supplemented with a GFP expression vector. Differentiation was induced 2 days later (D0). Cells were fixed at D0 or after four days of differentiation (D4) in 4% PFA, stained with an anti-desmin antibody (muscle cell marker), and nuclei were counterstained with DAPI. (*A*) The fusion index at D0 was calculated as the percentage of nuclei in GFP-positive cells with at least 2 nuclei. (*B*) The fusion index at D4 of differentiation was calculated as the percentage of nuclei in desmin-positive cells with at least 2 nuclei. (*C*) Representative images of desmin- and DAPI-labelled cells transfected with the control, syncytin-A, syncytin-B or myomaker vectors at D4. Data are the mean ± SEM (three independent experiments; * p<0.05, ** p<0.01, Student’s t-test).

To characterize further the role of the *syncytin* genes in myoblast fusion, we isolated mouse primary satellite cells and quantified the expression of both *syncytin-A* and–*B* in proliferating myoblasts, as well as during the process of cell differentiation and fusion (day 0, day 2 and day 4, see Fig 5A). Both genes were detected by quantitative RT-PCR and were found to be upregulated after 2–4 days of myogenic differentiation and myoblast fusion (when myotubes could be clearly observed), whereas the level of the housekeeping gene remained stable. These data suggest that both *syncytin* genes could be involved in this process. To investigate further their role in myoblast fusion, we knocked-down *syncytin-A* and–*B* expression by RNA-mediated interference (Fig 5B). After two days of differentiation, silencing of both *syncytin* genes resulted in a very significant inhibition of myoblast fusion as illustrated in Fig 5C, with a close to 50% decrease in the fusion index (Fig 5D). Analysis of the nuclei distribution in myoblast cells revealed a significantly higher number of mononucleated cells in cells transfected with either *syncytin-A* or *syncytin-B* siRNAs in comparison to cells transfected with a control siRNA (Fig 5E; * p<0.05, ** p<0.01, Student’s t-test). In addition, a significantly higher number of cells with >4 nuclei was observed in control cells in comparison to cells transfected with *syncytin-A* or *syncytin-B* siRNAs, with no myotube containing >8 nuclei in both cases. Combination of the two *syncytin* siRNAs had no additive effect (Fig 5D) suggesting that, at least *ex vivo*, the two *syncytins* are acting on similar–and most probably interactive- steps of myoblast fusion. Of note, reduction in fusion index mediated by siRNA targeted to *syncytins* remains smaller than that with a siRNA targeted to the *myomaker* gene (ref. 17 and Fig 5D), consistent with the notion that syncytins are only “add-on” contributors to the basal fusogenic activity associated with the non-syncytin genes. Yet both syncytins are able to trigger myoblast cell-cell fusion. This is illustrated in Fig 6, where transfection of C2C12 myoblasts with expression vectors for either syncytin-A or syncytin-B clearly triggered the fusion of myoblasts in their proliferative state (Fig 6A), and also enhanced the fusion normally triggered upon differentiation of the cells (Fig 6B and 6C). Altogether, the *ex vivo* data corroborate the *in vivo* data on syncytin-B, and further suggest that syncytin-A could also contribute to myoblast fusion.

### The role of *syncytin* genes in myoblast fusion extends to other mammals

**Fig 7:**
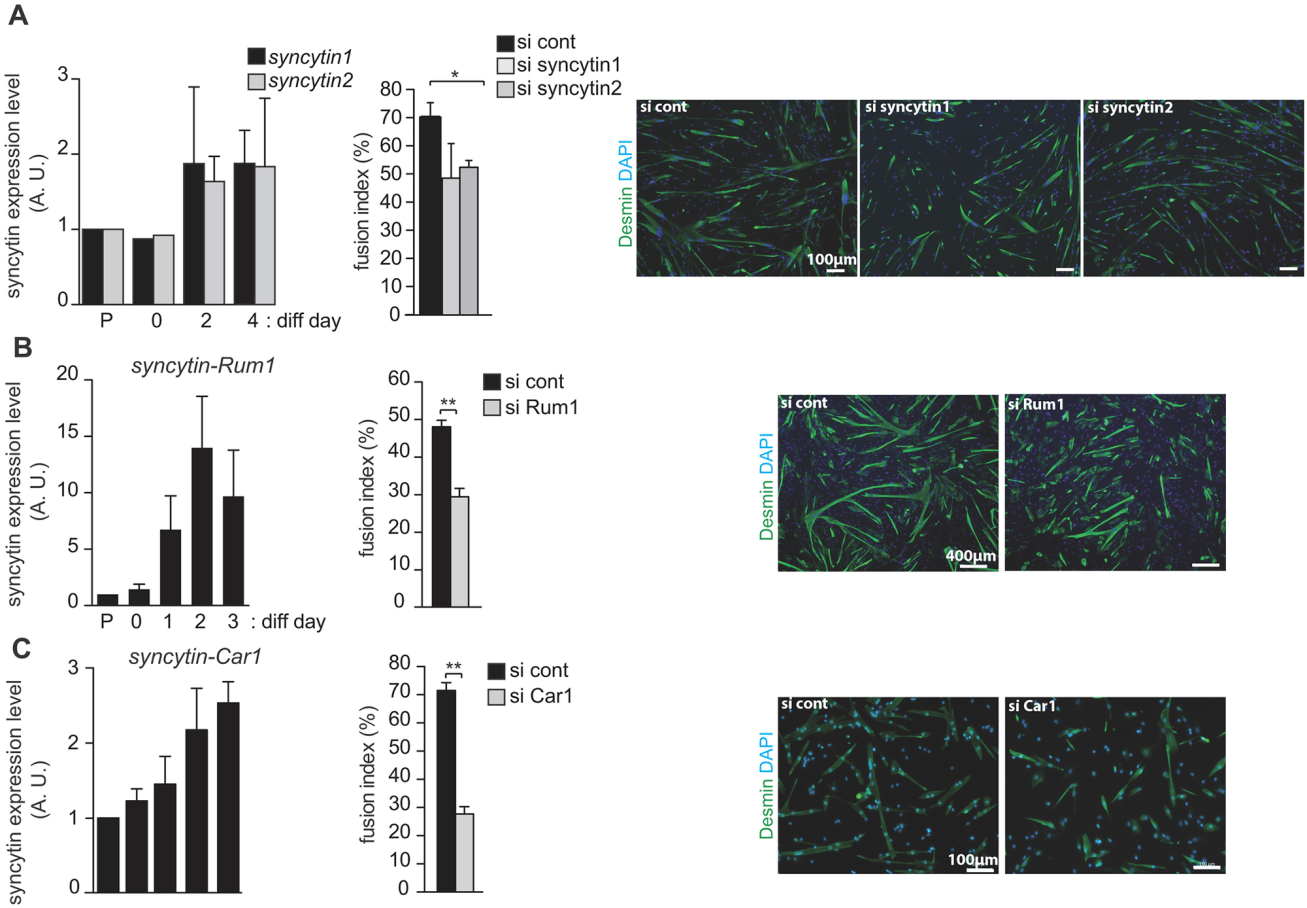
Role of *syncytins* in myoblasts from human, sheep and dog. Left panels: level of expression of the human *syncytin-1* and *-2* (*A*), the ruminant *syncytin-Rum1* (*B*) and the carnivore *syncytin-Car1* (*C*) genes (arbitrary units, A. U.) during proliferation and differentiation of primary myoblasts from human, sheep and dog, as determined by quantitative RT-PCR and normalized by the beta2-microglobulin, SDHA and PPIA housekeeping genes, respectively; the fold-change in gene expression was expressed relative to the values of proliferating myoblasts, arbitrarily settled to one. Middle and right panels: impact of siRNA-mediated knockdown of *syncytins* on *ex vivo* cell-cell fusion of myoblasts from human, sheep and dog, respectively. Cells were transfected with siRNA (control or specifically designed against the corresponding *syncytins*), and differentiation induced 2 days later. Following 2 days of differentiation, cells were fixed in 4% PFA, stained with an anti-desmin antibody (muscle cell marker), and nuclei were counterstained with DAPI. The fusion index (number of nuclei in cells with >2 nuclei per total number of nuclei) was determined (middle panels). Representative images of desmin-immunolabeled and DAPI-stained myoblasts transfected with either control or *syncytin*-specific siRNAs, respectively, are shown (right panels). Data are the mean ± SEM (at least three independent experiments; * p<0.05, ** p<0.01, Student’s t-test).

To determine if the effects observed in the mouse can be extrapolated to other placental mammals, we established primary cultures of myoblasts from the muscles of mammals where the cognate *syncytins* have been characterized. Accordingly, we obtained viable and fusion-prone myoblasts from human (primate) (Fig 7A), sheep (ruminant) (Fig 7B) and dog (carnivore) (Fig 7C). As illustrated in Fig 7, left panels, induction of each cognate *syncytin* gene could be observed during the process of cell differentiation and fusion, as similarly observed for the murine myoblasts (Fig 5), whereas the level of the housekeeping genes remained stable. Experiments were then carried out as above for the mouse myoblast cells, but using siRNAs specific for each of the corresponding *syncytins* (i.e., *syncytin-1* and *-2*, *syncytin-Rum1* and *syncytin-Car1* respectively). Accordingly, transfection of the cells with siRNA specific for each cognate *syncytin* clearly resulted in a significant (>20%) reduction in the measured fusion index (Fig 7, middle panels), also visible in the reduction in the number of large myotubes (Fig 7, right panels).

## Discussion

In this study, we address for the first time the role of the two murine *syncytin* genes (*syncytin-A* and–*B*, in myogenesis, both *in vivo* using a *syncytin-B* knockout mouse model, and *ex vivo* using primary cell cultures derived from isolated muscle satellite cells. We show that both *syncytin* genes are expressed *ex vivo* during the fusion of myoblast cells and that *syncytin-B* is expressed in regenerative fibers *in vivo* following cardiotoxin injury. Using *syncytin-A* and*–B* siRNAs, we demonstrate *ex vivo* that both *syncytin* genes contribute to myoblast cell fusion. In addition, using a knockout mouse model for *syncytin-B*, we show that impairment of *syncytin-B* leads to a reduction in muscle mass, that largely contributes to the observed reduction in body weight. This effect is male-specific, being not observed in females, that provide a clear-cut internal control. This decreased mass correlates with an observed reduction in several muscle parameters, including the muscle cross-sectional area, the individual myofiber cross-sectional area, and most importantly the number of nuclei per muscle fiber. Furthermore, we show that the total number of fibers per muscle is not altered in *syncytin-B* knockout mice, suggesting that *syncytin-B* activity is essentially involved in myofiber growth and not in the initiation of myogenesis. The reduction in the number of nuclei per myofiber would be consistent with such a role and with the fusogenic activity demonstrated in the *ex vivo* experiments. Finally, using a model of muscle regeneration after injury by cardiotoxin injection, we show that impairment of *syncytin-B* results in a delay in muscle regeneration, again only observed in males, consistent with the specific induction of *syncytin-B* expression observed in wild-type regenerating male myofibers. To summarize, the present data indicate that *syncytin-B* (and possibly *syncytin-A*, see below), participates in myoblast fusion, and contributes to muscle growth and regeneration in the mouse, in a sex-dependent manner.

This series of results raise several issues. One of the most obvious questions is related to the function of *syncytin-A*. Indeed, our cell fusion assays performed in C2C12 cells and in primary myoblasts strongly suggest that both *syncytins* have closely related activities, with similar effects of synA and synB siRNAs on myoblast fusion. This would fit with the known properties of these two genes, which have very similar activities at the placental level, each one being involved in the formation of a definite syncytiotrophoblast layer at the feto-maternal interface [8]. However a definitive characterization of the role of *syncytin-A* in myogenesis will require its conditional knock-out, specifically in the muscle, because homozygous null placentae are embryonic lethal. Along these lines, a mouse model obtained by crossing a lox-*syncytin-A* knock-in mouse with a Pax7-Cre-ERT2 mouse [23] is being developed, which should allow to generate a muscle-specific knock-out of *syncytin-A*, thus circumventing the detrimental effect of *syncytin-A* inactivation in placentation.

Another important issue concerns the generality of the contribution of *syncytin* genes to myogenesis. We have clearly demonstrated that not only the murine *syncytins* but also *syncytins* from ruminants, carnivores and humans contribute to myoblast fusion, at least in *ex vivo* assays. Similar conclusions were reached for human myoblasts [15, 24], where either siRNAs or an antibody targeting *Syncytin-1* inhibit cell fusion of cultured myoblasts. Although the physiological relevance of such assays can be questioned, they nevertheless strongly suggest that the effects observed for the murine genes might extend to all species possessing a *syncytin*. Indeed, in the present study, we tested all four species with identified *syncytins* for which we succeeded in establishing primary myoblasts prone to cell fusion (we could not isolate myoblasts from the rabbit, in which we had previously characterized *syncytin-Ory1*, [25]), and in all four cases the results were identical, with inhibition of fusion by the corresponding *syncytin* gene-specific siRNA. It is therefore tempting to speculate that *syncytins*, which have been captured in all placental mammals most probably primarily for their placental function, all contribute, as observed here *in vivo* in the mouse, to myogenesis. However, this hypothesis will remain difficult to demonstrate or invalidate unless, for instance, myoblast fusion is found to be independent of *syncytin*, in a species where the gene is present. At the molecular level, the myoblast fusion process is still an unresolved question, although it is clear that it involves a wide panel of genes (for a review see ref. [9, 12]). Some have been shown to be required for myogenesis *in vivo* (via the use of KO mice), such as the recently identified *myomaker* gene [17, 18], the contributing *galectin* gene [26], cell adhesion/signal transduction proteins [27] or a series of actin cytoskeletal remodeling factors (reviewed in [9, 28]). Yet, in all cases none of the identified genes are truly autonomous fusogens, but rather seem to be either co-factors, yet absolutely required for myogenesis, or partners of a still to be identified “universal” fusogen. Our data on myomaker transfection of C2C12 myoblasts (Fig 6) do not provide evidence for a direct fusogenic activity in the proliferating state (at variance with syncytin A and B). This result is consistent either with myomaker not being a bona fide fusogen or its fusion partner only being expressed upon myoblast differentiation. In the case of the presently identified *syncytins*, these are bona fide fusogens, a function inherited from the envelope gene of the ancestral retroviruses from which they were captured. Their contribution to myoblast fusion, as well as to cytotrophoblast or macrophage fusion, could in that case be directly due to their fusogenic activities.

An additional important question arises from the unexpected result on the sex-dependent effect of *syncytin-B in vivo*, in myogenesis after birth and muscle regeneration after injury. The data clearly indicate that the *syncytin-B* effect is essentially an “add-on” specific to the male, not taking place in the female, and resulting in an increased number of nuclei per muscle fiber (consequently associated with an increased muscle mass) and an increased rate of muscle regeneration after injury. Then a question arises as to the molecular origin of this uncovered male-dependence. Among the possible mechanisms, a hormone-dependent regulation of *syncytin* expression would of course fit the data, and could be analyzed by searching for binding sites within the *syncytin* gene promoter/enhancer for hormone–dependent factors. Along these lines, it is clear that testosterone and the androgen receptor (AR) would be appropriate effectors, which could be tested by hormone injection to wild-type mice, with or without prior castration, or even in mice knocked-out for the AR gene [29]. It is also possible that factors known to act as transcription factors in the placenta (among others GCM1 [30, 31]) could also act as *syncytin* activators in the muscle. Experiments are now in progress to tentatively elucidate these points. It is also noteworthy that testosterone and/or sustained physical activity (the latter known to generate microlesion-and-repair cycles) have been reported to result in muscle mass increase [24, 32–34], with, in some cases, indications for an increase in myonuclei number per fiber [35, 36]. Moreover, one of these studies shows that long-term endurance exercise in male humans results in an increase in *Syncytin-1* expression, among other genes, in muscle biopsies [24]. It will be interesting to carry out a similar study on female humans to determine whether *syncytin-1* induction is male-specific, or not. Indeed, such sex-specific effects could be a hint for a generalized male-dependence of *syncytin* expression and role in myogenesis among placental mammals. If so, it is likely that *syncytins* could then be responsible for the muscle sexual dimorphism observed in mammals, where in most cases the male has a higher body and muscle mass than the female.

To conclude, we propose as a working model that syncytins have been captured primarily as a placental gene, but that some expression in the muscle is taking place (possibly as a consequence of regulatory processes and transcription factors shared by placenta and muscle), and that a “collateral” effect of the capture of such genes -with a clear-cut fusogenic activity- is an add-on contribution to myogenesis in males of placental mammals.

## Methods

### Animal model, ethical agreement

Targeted mutagenesis of *syncytin-B* gene, located on chromosome 14, was described previously [8]. Briefly, the mouse syncytin-B ORF (carried by a single 1.8-kb exon) was deleted by homologous recombination using a strategy based on the Cre/LoxP recombination system for generating KO mice. Male and female mice were on a mixed 129/Sv C57BL/6 background. They were housed under controlled conditions at 24°C, and were allowed free access to food and water in full compliance with the French government animal welfare policy. All the experiments were approved by the Ethics Committee of the Gustave Roussy Institute.

### Experimentally induced muscle regeneration

Before manipulations mice were anaesthetized with an intraperitoneal injection of 0.1 ml per 10 g body weight of a solution containing 1 mg/ml xylazine (Bayer) and 10 mg/ml ketamine (Merial) per mouse. Then, to induce muscle injury, 35 μl of Cardiotoxin (Ctx) (12 μM; Latoxan) were injected in a single injection into *Tibialis Anterior* (TA) muscle of 8 week old mice. Muscle regeneration was followed at different times depending on the experiments.

### Histological and immunofluorescence analyses

Mouse *Tibialis anterior* (TA), *Extensor Digitorum Longus* (EDL) and Soleus (SOL) muscles were dissected, weighed and frozen in isopentane/liquid nitrogen baths. Cryopreserved tissues were then cut in 10 μm cryosections. Cryosections were cut in the same central region of the muscle for all analyzed animals. Muscle cryosections were fixed in 4% paraformaldehyde on ice for 8 min. Tissue sections were then saturated with 5% Goat serum 2% BSA solution for 30 min. Overnight immunolabeling for dystrophin (Leica Biosystems) and laminin (Sigma) was then performed at 4°C. Nuclei were counterstained with 4',6'-diamidino-2-phenylindole (DAPI) for 15 min at room temperature. Myofiber cross-sectional area (CSA) was measured from stained slides from randomly chosen fields using the ImageJ software (at least 150 myofibers were measured per condition).

### *In situ* hybridization (ISH)

Freshly collected muscles were fixed in 4% (wt/vol) paraformaldehyde and embedded in paraffin. Serial sections (7 μm) were used for ISH. For the *syncytin-B* gene, three PCR-amplified *syncytin-B* fragments of 414, 511, and 370 bp, respectively (primers listed in S1 Table) were cloned into pGEM-T Easy (Promega). *In vitro* synthesis of the antisense and sense riboprobes was performed with SP6 or T7 RNA polymerase and digoxigenin 11-UTP (Roche Applied Science) after cDNA template amplification. Sections were processed, hybridized at 42°C overnight with the pooled riboprobes, and incubated further overnight at 4°C with alkaline phosphatase-conjugated anti-digoxigenin antibody Fab fragments (Roche Applied Science). Staining was achieved with the nitroblue tetrazolium (NT) and 5-bromo-4-chloro-3-indolyl phosphate (BCIP) phosphatase alkaline substrates as indicated by the manufacturer (Roche Applied Science).

### Primary muscle cell culture and fusion assay

Mouse primary myoblasts (female mice) were isolated from TA muscles of 4 week old mice by enzymatic digestion as described [37]. Myoblasts were grown on collagen-coated dishes in Ham’s F10 (Life technologies) growth medium supplemented with 20% fetal bovine serum (FBS), bFGF (10 ng/ml, Invitrogen), penicillin (100 U/ml) and streptomycin (100 mg/ml). For differentiation, myoblasts were plated at 10^5^ cells per well in collagen-coated 24-well dishes and differentiation was induced by switching the growth medium to the differentiation medium DMEM (Life technologies) with 2% horse serum (Biowest) for different times, the day of the induction being referred to as D0.

Dog (male) and sheep (female) myoblasts were obtained from biopsies of biceps femoris. The biopsy was cut into small pieces, treated with collagenase (Worthington; 5 ml of collagenase per g of tissue) and incubated for 1 h at 37°C. Using a syringe and 18G needle, the mix was dissociated and filtered sequentially through 100 and 40 μm sieves. After centrifugation, the cellular pellet was suspended and in the case of dog myoblasts, cells were seeded in Myo1 medium (Hyclone) containing 20% FBS, gentamycin (25 μg/ml, Sigma), bFGF (10 ng/ml, Invitrogen) and dexamethasone (1 μM, Mylan). Sheep myoblasts were cultivated on collagen-coated dishes in DMEM medium (Life technologies) with 20% FBS, bFGF (10 ng/ml, Invitrogen), penicillin (100 U/ml) and streptomycin (100 mg/ml). For differentiation, growth medium was replaced with DMEM medium (Life technologies) supplemented with 2% horse serum (Life technologies).

Primary human myoblasts (female) were cultivated in proliferation medium (4 vol. of DMEM, 1 vol. of 199 medium, 20% FBS, gentamycin (50 mg/ml)). Differentiation was induced by replacing the proliferation medium by DMEM supplemented with insulin (10 mg/ml).

For desmin immunolabeling, cells were fixed for 10 min with 4% formaldehyde and rinsed with PBS. Cells were permeabilized for 10 min with PBS-0.1% Triton (Sigma) and blocked with PBS-5% BSA (Sigma) for 45 min before incubation with antibodies. Primary mouse anti-desmin anti-human antibodies (clone D33, Dako) were incubated overnight at 4°C and revealed using secondary Alexa 488 goat anti-mouse antibodies (life technologies) for 1h. Nuclei were stained with DAPI (4’,6’ diamidino-2-phenylindol) for 15 min at room temperature.

The fusion index was calculated as the number of nuclei within cells with ≥2 nuclei at day 3 of differentiation (at least 600 nuclei were counted for each condition).

### RNA interference

RNA interference was performed using Lipofectamine RNAi-MAX reagent (Invitrogen) according to the manufacturer recommendations. Cells were transfected 2 days before induction of differentiation. All siRNAs, including the siRNA control oligonucleotides, were synthesized by Dharmacon or Eurogentec. SiRNA sequences are listed in S1 Table.

### C2C12 cell culture

The C2C12 mouse myoblast cell line (ATCC: CRL1772) (female mice) was grown in DMEM (Dulbecco’s Modified Eagle medium, life technologies) supplemented with 10% fetal calf serum (FCS), 100 U/ml penicillin and 100 μg/ml streptomycin. Differentiation was induced by switching the growth medium to differentiation medium (DMEM with 2% horse serum (Biowest)) for four days.

### Plasmids and cell transfection

The GFP expression vector peGFP-C3 was purchased from BD Biosciences and the murine myomaker gene in pCMV-SPORT6.1 from Dharmacon. The control (phCMV-none) and syncytin-A and–B expression vectors were previously described [6]. Transfection of the C2C12 cells was performed using Lipofectamine LTX (ThermoFischer) according to the manufacturer’s protocol.

### Quantitative real-time RT-PCR

Total RNAs were extracted from mouse, sheep and human myoblast cells using Trizol according to the manufacturer’s instructions (Sigma Aldrich). Total RNAs were extracted from dog myoblasts using the nucleospin RNA XS kit (Macherey Nagel) according to the manufacturer instructions. The amount of extracted RNAs was evaluated using a NanoDrop spectrophotometer (Thermo Scientific, Wilmington, USA). Syncytin mRNA expression was determined by Real-time RT-PCR (RT-qPCR). Reverse transcription was performed on 500 ng of DNase-treated RNAs (Ambion) according to the manufacturer’s instructions. Quantitative PCR was performed on 5 μL of diluted (1:5) cDNA in a final volume of 25 μL using the SYBR-Green PCR Master Mix (Applied Biosystems) in an ABI PRISM 7000 sequence detection system. The parameters used were as follows: 2 min incubation at 50°C, 5 min at 95°C, followed by 40 cycles of repeated incubations at 95°C for 10 s and 60°C for 30 s. For each cDNA sample, duplicates were analyzed and data normalized to the housekeeping genes listed in S1 Table.

### Statistical analysis

Histological analysis of the muscle phenotype in 12 week old mice and the muscle regeneration in 8 week old mice was performed on at least 150 myofibers randomly chosen from three different tissue sections. The number of myonuclei was estimated by counting the number of myonuclei in at least 300 myofibers randomly chosen from three different tissue sections per condition. Statistical analyses were performed using the Mann-Whitney test. A p value <0.05 was considered as significant (* p<0.05, ** p<0.01). When the number of samples was <4, Student’s t-test was used and differences were considered significant when p <0.05 (* p<0.05, ** p<0.01). For the *ex vivo* fusion assays, according to the number of samples, a Mann-Whitney test (n = 4, for mouse and sheep myoblast cells) or a Student’s t-test (n = 3, for C2C12, human and dog myoblast cells) was used and differences were considered significant when p <0.05 (* p<0.05, ** p<0.01).

## Supporting Information

S1 Fig Muscle mass, muscle CSA and myofiber area of individual 12 week old SynB^-/-^ and WT mice with mention of the cross from which they originate. Individual EDL muscle mass *(A)*, muscle CSA *(B)* and myofiber area *(C)* from wild-type (+/+) and SynB KO (-/-), male (m) and female (f) mice. The crosses from which the mice originate were labelled by 4 to 5 different markers (blue square, green triangle, yellow circle, dark blue cross, red star). The values represented by the same marker in WT and SynB KO mice in the same graph correspond to that of individual mice from the same cross. The mean muscle mass, muscle CSA and myofiber area are indicated by horizontal black lines.
(TIF)

S2 Fig Simplified scheme of skeletal muscle structure. (TIF)

S3 Fig Myonuclear length and number of myonuclei per mm of myofiber in 12 week old SynB^-/-^ mice. (*A*) Measure of the average myonuclear length of TA muscles from WT (+/+) and SynB^-/-^ (-/-), male (m) and female (f) mice. Data are the mean ± SEM (at least 60 myonuclei measured for each type) (*B*) Anti-dystrophin and DAPI labelling of 10 μm longitudinal sections of optimum cutting temperature (OCT)-frozen TA muscles from 12 week old WT and SynB^-/-^ male mice (scale bar: 10 μm). (*C-E*) Quantification of the myonuclei number per mm of fiber length in SOL, EDL and TA muscles of WT and SynB^-/-^ male and female mice. Data are the mean ± SEM (4–6 mice analyzed per sex and per genotype; * p<0.05, ** p<0.01, Mann and Whitney test).
(TIF)

S1 Table List of primers. (DOCX)

S1 File Supplementary Methods. (DOCX)

## References

[1] Lavialle C, Cornelis G, Dupressoir A, Esnault C, Heidmann O, Vernochet C, et al Paleovirology of 'syncytins', retroviral env genes exapted for a role in placentation. Philosophical Transactions of the Royal Society B-Biological Sciences. 2013;368(1626). doi: ARTN 20120507 10.1098/rstb.2012.0507. WOS:000331222100010. PMC3758191 23938756

[2] Cornelis G, Heidmann O, Bernard-Stoecklin S, Reynaud K, Veron G, Mulot B, et al Ancestral capture of syncytin-Car1, a fusogenic endogenous retroviral envelope gene involved in placentation and conserved in Carnivora. Proc Natl Acad Sci U S A. 2012;109(7):E432–41. 10.1073/pnas.1115346109 22308384 PMC3289388

[3] Cornelis G, Heidmann O, Degrelle SA, Vernochet C, Lavialle C, Letzelter C, et al Captured retroviral envelope syncytin gene associated with the unique placental structure of higher ruminants. Proc Natl Acad Sci U S A. 2013;110(9):E828–37. 10.1073/pnas.1215787110 23401540 PMC3587263

[4] Cornelis G, Vernochet C, Carradec Q, Souquere S, Mulot B, Catzeflis F, et al Retroviral envelope gene captures and syncytin exaptation for placentation in marsupials. Proc Natl Acad Sci U S A. 2015;112(5):E487–96. 10.1073/pnas.1417000112 25605903 PMC4321253

[5] Cornelis G, Vernochet C, Malicorne S, Souquere S, Tzika AC, Goodman SM, et al Retroviral envelope syncytin capture in an ancestrally diverged mammalian clade for placentation in the primitive Afrotherian tenrecs. Proc Natl Acad Sci U S A. 2014;111(41):E4332–41. 10.1073/pnas.1412268111 25267646 PMC4205661

[6] Dupressoir A, Marceau G, Vernochet C, Benit L, Kanellopoulos C, Sapin V, et al Syncytin-A and syncytin-B, two fusogenic placenta-specific murine envelope genes of retroviral origin conserved in Muridae. Proc Natl Acad Sci U S A. 2005;102(3):725–30. 10.1073/pnas.0406509102 15644441 PMC545540

[7] Dupressoir A, Vernochet C, Bawa O, Harper F, Pierron G, Opolon P, et al Syncytin-A knockout mice demonstrate the critical role in placentation of a fusogenic, endogenous retrovirus-derived, envelope gene. Proc Natl Acad Sci U S A. 2009;106(29):12127–32. 10.1073/pnas.0902925106 19564597 PMC2715540

[8] Dupressoir A, Vernochet C, Harper F, Guegan J, Dessen P, Pierron G, et al A pair of co-opted retroviral envelope syncytin genes is required for formation of the two-layered murine placental syncytiotrophoblast. Proc Natl Acad Sci U S A. 2011;108(46):E1164–73. 10.1073/pnas.1112304108 22032925 PMC3219115

[9] Abmayr SM, Pavlath GK. Myoblast fusion: lessons from flies and mice. Development. 2012;139(4):641–56. 10.1242/dev.068353 22274696 PMC3265056

[10] Bentzinger CF, Wang YX, Rudnicki MA. Building muscle: molecular regulation of myogenesis. Cold Spring Harb Perspect Biol. 2012;4(2). 10.1101/cshperspect.a008342 22300977 PMC3281568

[11] Buckingham M. Myogenic progenitor cells and skeletal myogenesis in vertebrates. Curr Opin Genet Dev. 2006;16(5):525–32. 10.1016/j.gde.2006.08.008 . 16930987

[12] Buckingham M, Rigby PW. Gene regulatory networks and transcriptional mechanisms that control myogenesis. Dev Cell. 2014;28(3):225–38. 10.1016/j.devcel.2013.12.020 . 24525185

[13] Chen EH, Olson EN. Unveiling the mechanisms of cell-cell fusion. Science. 2005;308(5720):369–73. 10.1126/science.1104799 . 15831748

[14] Soe K, Andersen TL, Hobolt-Pedersen AS, Bjerregaard B, Larsson LI, Delaisse JM. Involvement of human endogenous retroviral syncytin-1 in human osteoclast fusion. Bone. 2011;48(4):837–46. 10.1016/j.bone.2010.11.011 . 21111077

[15] Bjerregard B, Ziomkiewicz I, Schulz A, Larsson LI. Syncytin-1 in differentiating human myoblasts: relationship to caveolin-3 and myogenin. Cell Tissue Res. 2014;357(1):355–62. 10.1007/s00441-014-1930-9 . 24902667

[16] Yin H, Price F, Rudnicki MA. Satellite cells and the muscle stem cell niche. Physiol Rev. 2013;93(1):23–67. 10.1152/physrev.00043.2011 23303905 PMC4073943

[17] Millay DP, Sutherland LB, Bassel-Duby R, Olson EN. Myomaker is essential for muscle regeneration. Genes Dev. 2014;28(15):1641–6. 10.1101/gad.247205.114 25085416 PMC4117939

[18] Millay DP, O'Rourke JR, Sutherland LB, Bezprozvannaya S, Shelton JM, Bassel-Duby R, et al Myomaker is a membrane activator of myoblast fusion and muscle formation. Nature. 2013;499(7458):301–5. 10.1038/nature12343 23868259 PMC3739301

[19] Keefe AC, Lawson JA, Flygare SD, Fox ZD, Colasanto MP, Mathew SJ, et al Muscle stem cells contribute to myofibres in sedentary adult mice. Nat Commun. 2015;6:7087 Epub 2015/05/15. ncomms8087 [pii] 10.1038/ncomms8087 25971691 PMC4435732

[20] Darr KC, Schultz E. Hindlimb suspension suppresses muscle growth and satellite cell proliferation. J Appl Physiol (1985). 1989;67(5):1827–34. . 2600016 10.1152/jappl.1989.67.5.1827

[21] Mozdziak PE, Pulvermacher PM, Schultz E. Unloading of juvenile muscle results in a reduced muscle size 9 wk after reloading. J Appl Physiol (1985). 2000;88(1):158–64. . 10642376 10.1152/jappl.2000.88.1.158

[22] Rosenblatt JD, Parry DJ. Adaptation of rat extensor digitorum longus muscle to gamma irradiation and overload. Pflugers Arch. 1993;423(3–4):255–64. . 8321629 10.1007/BF00374404

[23] Lepper C, Conway SJ, Fan CM. Adult satellite cells and embryonic muscle progenitors have distinct genetic requirements. Nature. 2009;460(7255):627–U94. 10.1038/nature08209. WOS:000268454300050. 19554048 PMC2767162

[24] Frese S, Ruebner M, Suhr F, Konou TM, Tappe KA, Toigo M, et al Long-Term Endurance Exercise in Humans Stimulates Cell Fusion of Myoblasts along with Fusogenic Endogenous Retroviral Genes In Vivo. PLoS One. 2015;10(7):e0132099 Epub 2015/07/15. 10.1371/journal.pone.0132099 PONE-D-15-02026 [pii]. 26154387 PMC4495930

[25] Heidmann O, Vernochet C, Dupressoir A, Heidmann T. Identification of an endogenous retroviral envelope gene with fusogenic activity and placenta-specific expression in the rabbit: a new "syncytin" in a third order of mammals. Retrovirology. 2009;6:107 10.1186/1742-4690-6-107 19943933 PMC2789053

[26] Georgiadis V, Stewart HJ, Pollard HJ, Tavsanoglu Y, Prasad R, Horwood J, et al Lack of galectin-1 results in defects in myoblast fusion and muscle regeneration. Dev Dyn. 2007;236(4):1014–24. 10.1002/dvdy.21123 . 17366633

[27] Hamoud N, Tran V, Croteau LP, Kania A, Cote JF. G-protein coupled receptor BAI3 promotes myoblast fusion in vertebrates. Proc Natl Acad Sci U S A. 2014;111(10):3745–50. 10.1073/pnas.1313886111 24567399 PMC3956190

[28] Shilagardi K, Li S, Luo F, Marikar F, Duan R, Jin P, et al Actin-propelled invasive membrane protrusions promote fusogenic protein engagement during cell-cell fusion. Science. 2013;340(6130):359–63. 10.1126/science.1234781 23470732 PMC3631436

[29] MacLean HE, Chiu WS, Notini AJ, Axell AM, Davey RA, McManus JF, et al Impaired skeletal muscle development and function in male, but not female, genomic androgen receptor knockout mice. FASEB J. 2008;22(8):2676–89. 10.1096/fj.08-105726 . 18390925

[30] Anson-Cartwright L, Dawson K, Holmyard D, Fisher SJ, Lazzarini RA, Cross JC. The glial cells missing-1 protein is essential for branching morphogenesis in the chorioallantoic placenta. Nat Genet. 2000;25(3):311–4. 10.1038/77076 . 10888880

[31] Simmons DG, Natale DR, Begay V, Hughes M, Leutz A, Cross JC. Early patterning of the chorion leads to the trilaminar trophoblast cell structure in the placental labyrinth. Development. 2008;135(12):2083–91. 10.1242/dev.020099 18448564 PMC3159581

[32] Kadi F, Thornell LE. Concomitant increases in myonuclear and satellite cell content in female trapezius muscle following strength training. Histochemistry and Cell Biology. 2000;113(2):99–103. 10.1007/s004180050012. WOS:000086163600004. 10766262

[33] Serra C, Tangherlini F, Rudy S, Lee D, Toraldo G, Sandor NL, et al Testosterone improves the regeneration of old and young mouse skeletal muscle. J Gerontol A Biol Sci Med Sci. 2013;68(1):17–26. 10.1093/gerona/gls083 22499765 PMC3598367

[34] Sinha-Hikim I, Cornford M, Gaytan H, Lee ML, Bhasin S. Effects of testosterone supplementation on skeletal muscle fiber hypertrophy and satellite cells in community-dwelling older men. J Clin Endocrinol Metab. 2006;91(8):3024–33. 10.1210/jc.2006-0357 . 16705073

[35] Joubert Y, Tobin C. Satellite cell proliferation and increase in the number of myonuclei induced by testosterone in the levator ani muscle of the adult female rat. Dev Biol. 1989;131(2):550–7. . 2912808 10.1016/s0012-1606(89)80025-9

[36] Sinha-Hikim I, Roth SM, Lee MI, Bhasin S. Testosterone-induced muscle hypertrophy is associated with an increase in satellite cell number in healthy, young men. Am J Physiol Endocrinol Metab. 2003;285(1):E197–205. 10.1152/ajpendo.00370.2002 . 12670837

[37] Rando TA, Blau HM. Primary mouse myoblast purification, characterization, and transplantation for cell-mediated gene therapy. J Cell Biol. 1994;125(6):1275–87. 8207057 10.1083/jcb.125.6.1275 PMC2290930

