# Supplementary figures and images for: Genetic Evidence That Captured Retroviral Envelope syncytins Contribute to Myoblast Fusion and Muscle Sexual Dimorphism in Mice
Source: PLoS Genet. 2016 Sep 2;12(9):e1006289. doi: 10.1371/journal.pgen.1006289 (PMC5010199; doi:10.1371/journal.pgen.1006289)

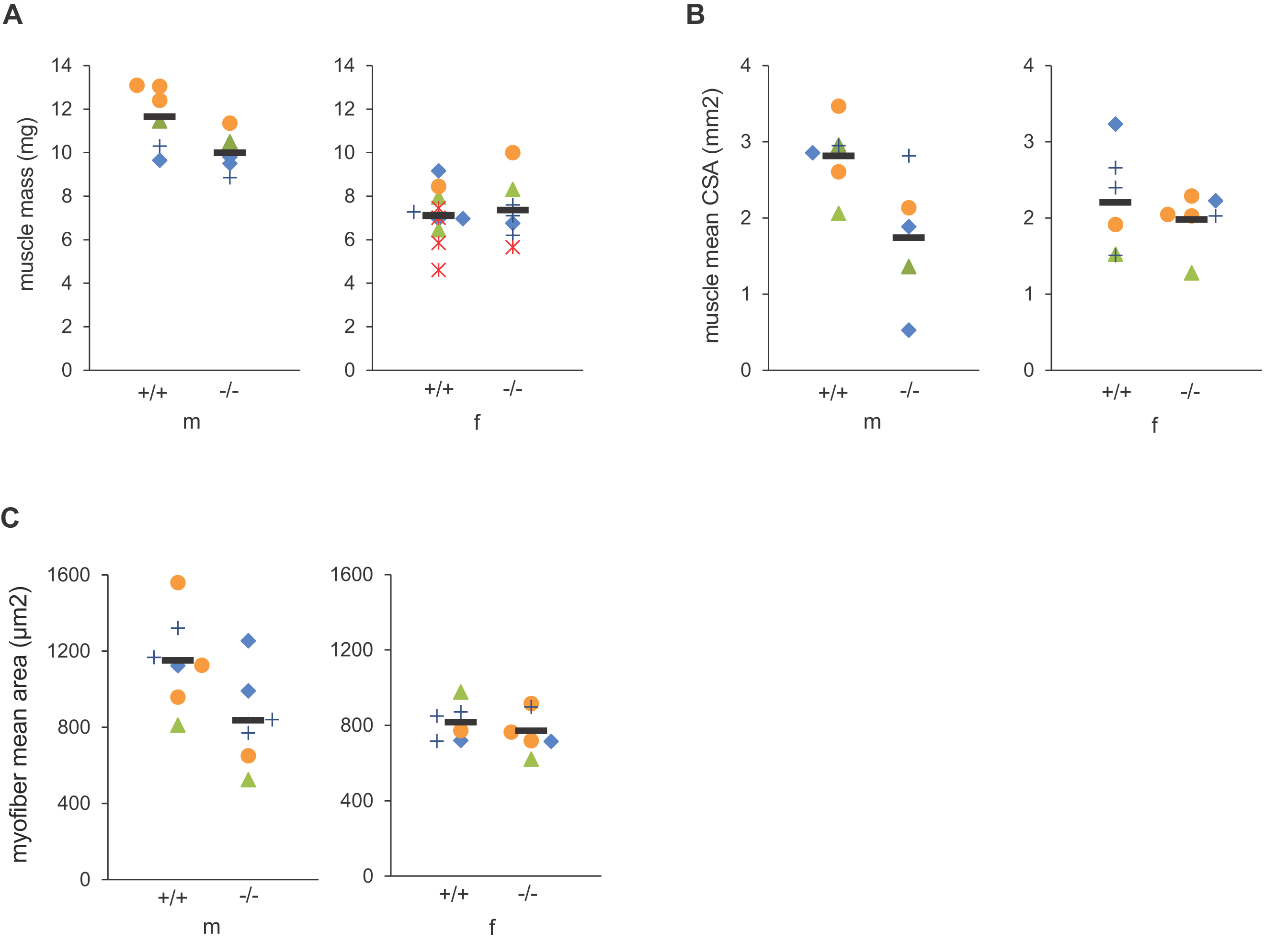

Supplement: S1 Fig — Individual EDL muscle mass (A), muscle CSA (B) and myofiber area (C) from wild-type (+/+) and SynB KO (-/-), male (m) and female (f) mice. The crosses from which the mice originate were labelled by 4 to 5 different markers (blue square, green triangle, yellow circle, dark blue cross, red star). The values represented by the same marker in WT and SynB KO mice in the same graph correspond to that of individual mice from the same cross. The mean muscle mass, muscle CSA and myofiber area are indicated by horizontal black lines. (TIF) [file pgen.1006289.s001.tif]

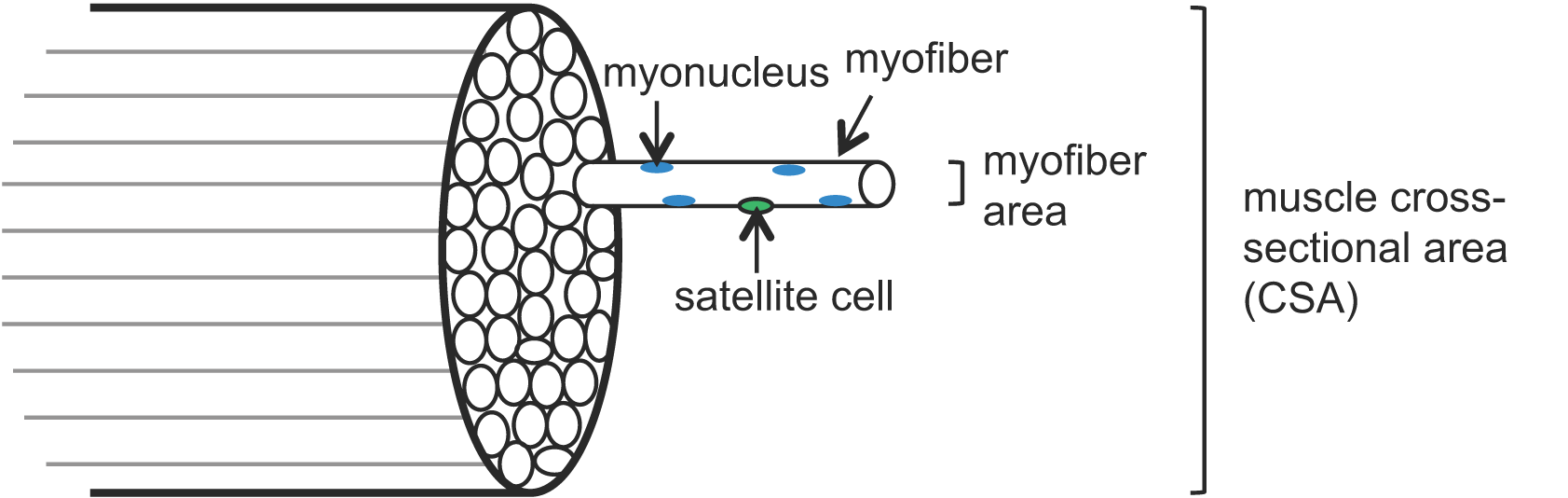

Supplement: S2 Fig — (TIF) [file pgen.1006289.s002.tif]

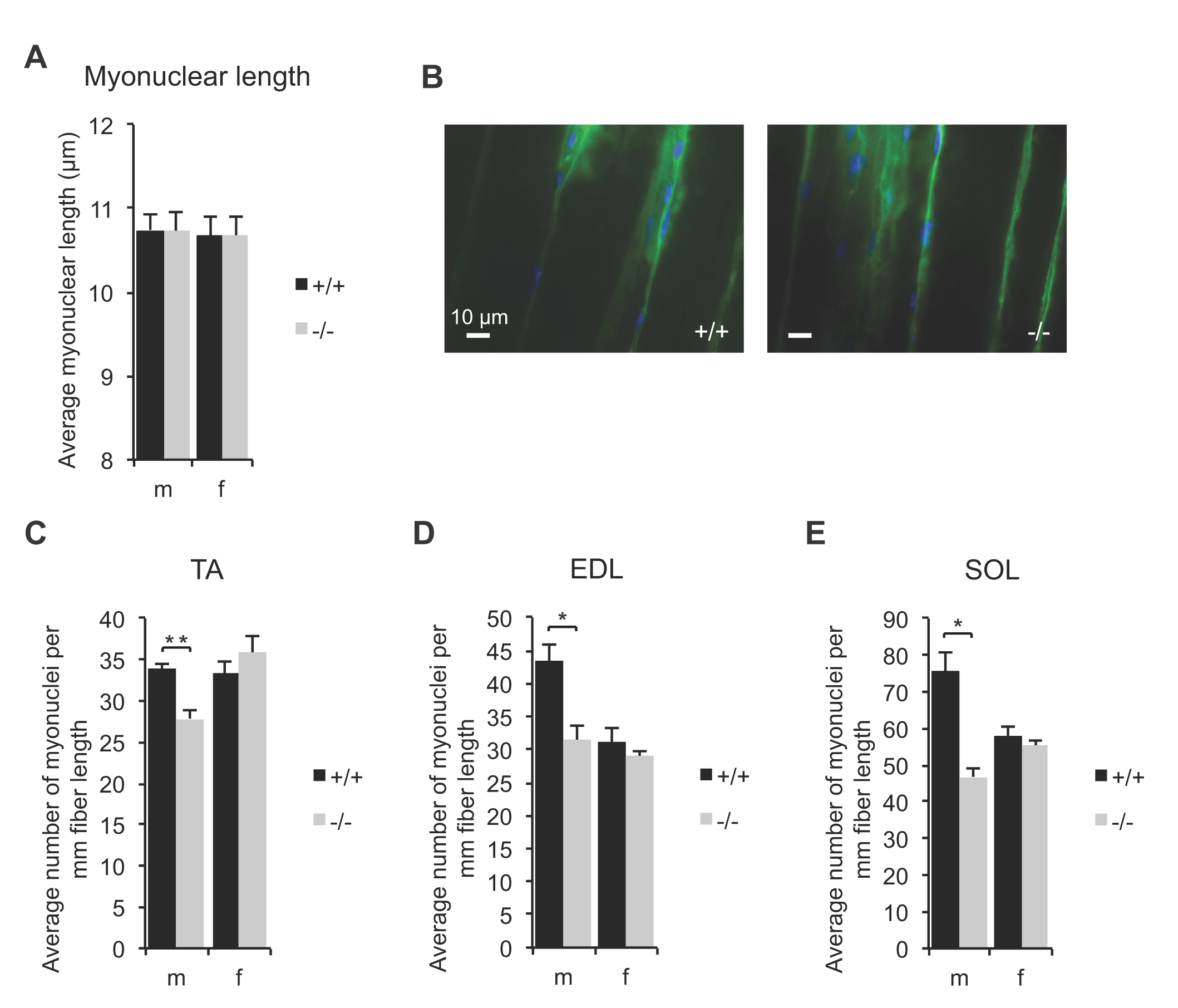

Supplement: S3 Fig — (A) Measure of the average myonuclear length of TA muscles from WT (+/+) and SynB-/- (-/-), male (m) and female (f) mice. Data are the mean ± SEM (at least 60 myonuclei measured for each type) (B) Anti-dystrophin and DAPI labelling of 10 μm longitudinal sections of optimum cutting temperature (OCT)-frozen TA muscles from 12 week old WT and SynB-/- male mice (scale bar: 10 μm). (C-E) Quantification of the myonuclei number per mm of fiber length in SOL, EDL and TA muscles of WT and SynB-/- male and female mice. Data are the mean ± SEM (4–6 mice analyzed per sex and per genotype; * p<0.05, ** p<0.01, Mann and Whitney test). (TIF) [file pgen.1006289.s003.tif]
