# Supplementary material for: Genetic Evidence That Captured Retroviral Envelope syncytins Contribute to Myoblast Fusion and Muscle Sexual Dimorphism in Mice
Source: PLoS Genet. 2016 Sep 2;12(9):e1006289. doi: 10.1371/journal.pgen.1006289 (PMC5010199; doi:10.1371/journal.pgen.1006289)
Supplement: S1 Table — (DOCX) [file pgen.1006289.s004.docx]

**S1 Table.** **List of primers**

Primer names Primer sequences

RT-qPCR

syncytin-A F 5’ TACTCCTGCCCGATAGATGA 3’

syncytin-A R 5’ CCGTTTTTCTTAACAGTGGGT 3’

syncytin-B F 5’ CCACCACCCATACGTTCAAA 3’

syncytin-B R 5’ GGTTATAGCAGGTGCCGAAG 3’

syncytin-Car1 F 5’ TATGGGACGAATTTATGTGGCT 3’

syncytin-Car1 R 5’ CCAATCAGTAGCAAACCAGTCA 3’

syncytin-Rum1 F 5’ CTGGGTAAGCGACTCCTG 3’

syncytin-Rum1 R 5’ AATTGGAGGAGTTGTGGAA 3’

syncytin-1 F 5’ CCCCATCGTATAGGAGTCTT 3’

syncytin-1 R 5’ CCCCATCAGACATACCAGTT 3’

syncytin-2 F 5’ GCCTGCAAATAGTCTTCTTT 3’

syncytin-2 R 5’ ATAGGGGCTATTCCCATTAG 3’

PPIA F 5’ GTCAACCCCATCGTGTTTTT 3’

PPIA R 5’ CTGCTGTCTTGGGAACTTTGTC 3’

SDHA F 5’ GCAGAACCTGATGCTTTGTG 3’

SDHA R 5’ CGTAGGAGAGCGTGTGCTT 3’

Rpl0 F 5’ GAGGACCTCACTGAGATTCGG 3’

Rpl0 R 5’ TTCTGAGCTGGCACAGTGAC 3’

b2m F 5’ CTCTCTTTCTGGCCTGGAGG 3’

b2m R 5’ TGCTGGATGACGTGAGTAAACC 3’

**In situ hybridization probe synthesis**

syncytin-B-ISH-F1 5’ GTGATACCAACTCTTCCCTGT 3’

syncytin-B-ISH-R1 5’ CATATGCTGAGACCTTGAAAA 3’

syncytin-B-ISH-F2 5’ TTTCACCTTCTACCAGTCATG 3’

syncytin-B-ISH-R2 5’ AGGCAGATGTAAGTCGATGTT 3’

syncytin-B-ISH-F3 5’ CTCGACTCTCTATTTCCAACA 3’

syncytin-B-ISH-R3 5’ TAGGCAGTTCAGAAGACAAGA 3’

**siRNA**

syncytin-1.1 5’ CCUGAACAAUGGAACAACU 3’

syncytin-1.2 5’ CCUUGCAAGAUCAACUUAA 3’

syncytin-1.3 5’ CCUCUAGCAGCUAUAAUAU 3’

syncytin-2.1 5’ GGAGGGCAAUCCAUUUCAU 3’

syncytin-2.2 5’ CCAACAGACUUACCAAACA 3’

syncytin-2.3 5’ CACCAUGGCUAAAGCCUUA 3’

syncytin-A.1 5’ GGUUAGGCAUCGCGGGUAU 3’

syncytin-A.2 5’ UCACUACUCCUGCCCGAUA 3’

syncytin-A.3 5’ GGCGGUAUCACUCCAAAUA 3’

syncytin-A.4 5’ GAAUAUGAACCAACUGUUA 3’

syncytin-B.1 5’ UGAACUGCCUAACCCGUUU 3’

syncytin-B.2 5’ GCAACUACACCAUGCAUAU 3’

syncytin-B.3 5’ GCUCGCAGGUGUUGUUCUA 3’

syncytin-B.4 5’ CCUAUCCGCUCUCCCAUUA 3’

syncytin-Car1.1 5’ GGAUGGCCUUAGACAUAAU 3’

syncytin- Car1.2 5’ CUGGCAUUAUUUACACCAA 3’

syncytin- Car1.3 5’ CUGGUAUGAUCACCUGGUA 3’

syncytin-Rum1.1 5’ CGCUAGUUAUGCAGCAUUA 3’

syncytin- Rum1.2 5’ GUCGAAUGUUGUGUAUAUA 3’

syncytin- Rum1.3 5’ CCACGAGCUUAUUGAUUAU 3’

myomaker-1 5’ UGACCAUUGCUGUGCGGAU 3’

myomaker-2 5’ AGGACUGGGACUACACUUA 3’

myomaker-3 5’ GCAUGUGGGUCUGCGUGAU 3’

myomaker-4 5’ CAAAGUGGCUACAGAAGAU 3’
