# Supplementary material for: Genetic Evidence That Captured Retroviral Envelope syncytins Contribute to Myoblast Fusion and Muscle Sexual Dimorphism in Mice
Source: PLoS Genet. 2016 Sep 2;12(9):e1006289. doi: 10.1371/journal.pgen.1006289 (PMC5010199; doi:10.1371/journal.pgen.1006289)
Supplement: S1 File — (DOCX) [file pgen.1006289.s005.docx]

**S1 File. Supplementary Methods**

**Quantification of myonuclear length and number of myonuclei per mm of fiber length:**

Optimum cutting temperature (OCT)-frozen TA muscles from 12 weeks old WT and SynB^-/-^ male and female mice were longitudinally cut in 10 μm cryosections. Muscle cryosections were fixed in 4% paraformaldehyde on ice for 8 min. Tissue sections were then saturated with 5% Goat serum 2% BSA solution for 30 min. Overnight immunolabeling for dystrophin (Leica Biosystems) was then performed at 4°C. Nuclei were counterstained with 4',6'-diamidino-2-phenylindole (DAPI) for 15min at room temperature. Myonuclear length was measured from stained slides from randomly chosen fields using the ImageJ software (at least 60 myonuclei were measured per condition). The number of myonuclei per mm of fiber length was calculated according to the methodology described in [19] using the equation (*i*): x = N*L/(l_m_+d), where x is the number of myonuclei per myofiber in a fiber segment of length L (1 mm in our case), N is the number of myonuclei in cross-section, l_m_ is the average myonuclear length of WT or SynB^-/-^, male or female TA muscles (average myonuclear length for TA was used for TA, EDL and SOL) and d is the thickness of the section (10 µm in our case).
